# Rodents Helminth Parasites in Different Region of Iran

**Published:** 2018

**Authors:** Nona MORADPOUR, Hassan BORJI, Jamshid DARVISH, Ali MOSHAVERINIA, Ahmad MAHMOUDI

**Affiliations:** 1. Rodentology Research Department, Institute of Applied Zoology, Faculty of Science, Ferdowsi University of Mashhad, Mashhad, Iran; 2. Dept. of Pathobiology, School of Veterinary Medicine, Ferdowsi University of Mashhad, Mashhad, Iran; 3. Dept. of Biology, Faculty of Science, Ferdowsi University of Mashhad, Mashhad, Iran

**Keywords:** Rodent, Helminth parasites, Iran

## Abstract

**Background:**

Climate condition is expected to have significant in rodents’ diversity and in the seasonal pattern of diseases carried by different rodents. In an effort to aid in the study of the biodiversity of parasites of rodents in different climate zoon we examined climate patterns in the parasite assemblages of different rodents from Mar 2015 to Feb 2016.

**Methods:**

Of 253 captured rodents in three climate zone of Iran, thirteen species of rodents were recognized. Rodents included *Mus musculus*, *Microtus*, *Apodemus witherbyi*, *Calomyscus elburzensis*, *Meriones libycus, Tatera indica*, *Alactaga elater,* and *Arvicola amphibius*. Trapped rodents humanely sacrificed and the gastrointestinal and respiratory tracts were removed and examined to identify parasitic helminths. Parasites were identified using key morphological characteristics.

**Results:**

Of 253 rodents examined, 109 (43.08%) were positive for helminth infection including *Syphacia obvelata* (20.1%), *Aspicularis tetraptera* (9.9%), *Trichuris muris* (0.3%), *Capillaria* sp. (0.3%), *Physaloptera* sp. (0.7%), *Gongylonema* sp. (1.1%), *Nippostrongylus brasiliensis* (6.7%) *Heligmosomoides polygyrus* (4.3%) *Hymenolepis diminuta* (3.1%), *H. nana* (0.8%), *Cysticercus fasciolaris,* (2.7%), *Mesocestoides* sp. larva (0.3%) and *Moniliformis moniliformis* (0.3%). *Notocotylus neyrai* was the only species of Trematoda isolated from water vole (*Arvicola amphibius*) for the first time in Iran.

**Conclusion:**

Some rodents are omnivorous, showing high predisposition to helminths parasites consequently, they harbor some species of parasites which are potentially zoonotic or may serve as vectors of important zoonotic pathogens. Therefore, the potential health hazard of these species needs to be considered to prevent infectivity of humans.

## Introduction

Owing to the different ecological conditions, divers topography and being as a corridor for the faunal exchange, Iran deserves to be account as a hotspot for rodents (1). It is not difficult to expect such level of diversity for their associated organisms, e.g. ecto and endoparasites. Seasonal pattern and temporal distribution of rodents- borne diseases are affected by climate conditions (2, 3).

Rodents harbor a number of endoparasites thus posing threats to health of human beings who live in close nearness to rodent populations. The parasitic eggs are passed out in rodent dung on agricultural fields, stored grains and in a range of edible commodities in houses and accordingly responsible for spread of the disease. Their capacity to act as a vector is seriously improved due to their physiological similarities which they distribute with humans (4). Consequently, increased rodent population in a region could be directly related to increased zoonotic diseases in human population (5). In spite of previous studies on helminth parasites of rodents in Iran (6–12), there is, however, less knowledge about helminths’ infection, their distribution, and diversity in the most regions of Iran. In short, most parts of previous works were only local investigations on North and South West of Iran (8, 10). Therefore, magnitude of our ignorance in country scale depends mostly on the small range of studies and the limited number of analyzed species.

We aimed to understand the distribution of rodent’s helminth parasites and their public health risks in different region of Iran. We trapped rodents from different regions of Iran verity of habitats.

## Materials and Methods

### Study area

Iran with an area of 1648000 km^2^, ranks eighteenth in size among the countries in the world. It is a vast country with different climate zoon in West Asia.

**Fig. 1:**
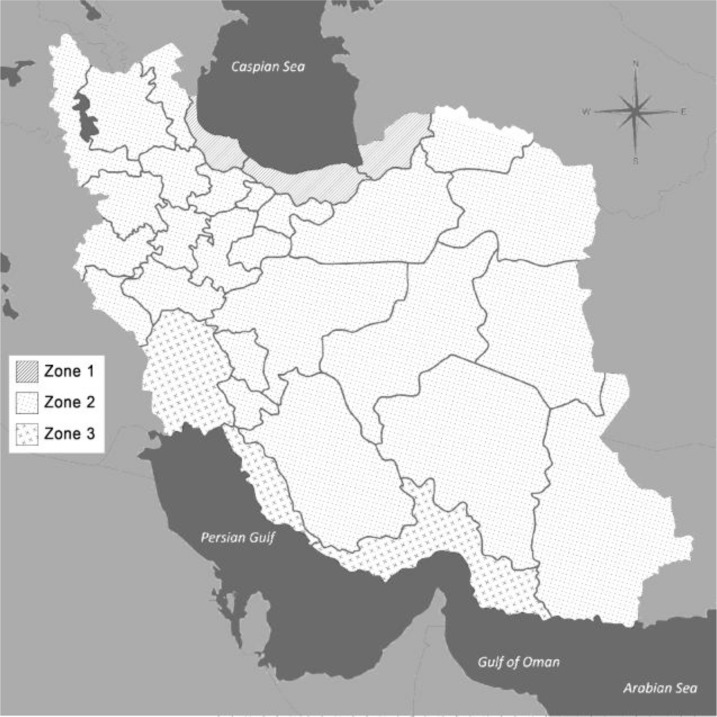
Map of Iran showing three different climatic regions where rodents were collected

Rodents were collected using live traps from different regions of Iran, where climate zones could be classified into three different types as follows (Fig. 1).

The first climate zone corresponds to the area with high humidity and wet habitats along southern margins of the Caspian Sea, which we performed sampling from Gorgan and Ali Abad-e Katoul (Golestan Province). Most sampling were carried out from the second climate zone with cold and high mountainous areas from eastern and western Iran such as, Cheshme Hesar and Zoshk (Khorasan Razavi), Kakhk (South Khorasan), Darekesh and Bojnord (North Khorasan), Yasouj (Kohgiluyeh & Boyer Ahmad), Shahmirzad (Semnam), Shahr-e Kord (Chaharmahal & Bakhtiari), Shiraz (Fars), Zanjan and Sistan & Baluchistan. The third climate zone corresponds to the arid and semi-arid regions, Ahvaz (Khuzestan).

### Animals

Overall, 253 rodents belonging to the thirteen species were trapped from Mar 2015 to Feb 2016. Traps were set at outdoors in agricultural, horticultural and animal farm and other suitable places in both urban and rural. Trapped rodents were transferred to Rodentology Research Department, Institute of Applied Zoology, Ferdowsi University of Mashhad and anesthetized under chloroform inhalation. Identification of the species was done based on available body of evidence (13–15).

The study was approved by Ethics committee of the university.

After dissection, rodent skin was removed and the body cavity was slitten open from the throat to the anus, revealing the esophagus, stomach, intestine, liver and urinary bladder. The esophagus, stomach, intestine, and liver were placed and opened longitudinally in individual Petri dishes containing 0.95% mammalian saline solution (MSS). The contents were examined both with the naked eye and under a stereoscope microscope and helminths were recovered and fixed in 70% alcohol.

Tapeworms and flukes were allowed to relax in tap water, fixed in 70% alcohol, pressed between two glass plates and stained with alum-carmine. Nematode parasites were fixed in 70% ethanol and cleared in lactophenol. Isolated parasites were identified using several taxonomic keys (16).

Abdominal cavity was examined for presence of metacestodes, cysts and livers with *Cysticercus fasciolaris* parasitic larval cysts were collected in normal physiological saline.

## Results

Altogether, 253 rodents were captured, of which the two genera *Mus* and *Microtus* were the largest groups. All *Mus* specimens were identified as *Mus musculus* (76 = 30%), while genus *Microtus* (70= 27.6%) consisted of the six different species; *M. paradoxus*, *M. irani, M. transcaspicus*, *M. qazvinensis*, *M. mystacinus* and *M. socialis*. The third and fourth abundant groups (51= 20.1%) were belong to the genus *Apodemus* (*A. witherbyi*), and *Calomyscus* (*C. elburzensis*) (21= 8.3%), respectively. Other species of rodents were captured less frequently: 13 specimens (=5.1%) as *Meriones libycus,* 15 specimens (=5.9%) as *Tatera indica,* six specimens (=2.3%) as *Alactaga elater* and only one sample (0.4%) as *Arvicola amphibius.*

Frequency distributions for the thirteen rodent species sampled from different regions of Iran were shown in Table 1.

**Table 1:** Distribution of the collected rodents’ species throughout the 11 provinces of Iran, Abbreviations refer to each locality are as follows: **NK**=North Khorasan, **RK**=Razavi Khorasan, **SK**=South Khorasan, **KB**= Kohgiluyeh & Boyer Ahmad, **KZ**=Kuzestan, **SE**=Semnan, **CB**=Chaharmahal & Bakhtiari, **SB**= Sistan & Baluchestan, **GO**=Golestan, **FA**=Shiraz, **ZA**=Zanjan

***Species*** **Locality**	***M. musculus***	***Microtus sp.***	***C. elburzensis***	***A.witherbyi***	***M. libycus***	***T. indica***	***A.a elater***	***A. amphibius***	***Total***
NK	18	16	-	12	2	-	-	-	48
Rk	4	-	3	10	-	-	4	-	21
Sk	23	-	-	-	-	2	-	-	25
KB	5	-	5	13	2	-	-	-	25
KZ	-	-	3	-	5	6	-	-	14
SE	-	6	-	2	-	-	-	-	8
CB	-	15	6	-	-		-	-	21
SB	7	-	2	-	2	7	-	-	18
GO	15	18	-	8	-	-	-	1	42
FA	2	13	2	2	-	-	-	-	19
ZA	2	2	-	4	2	-	2	-	12
Total	76	70	21	51	13	15	6	1	253

The infection rate of these species with helminths is shown in Table 2 shows that 43.08% of examined animals were infected at least with one species of parasites, whereas, Table 3 and 4 are correspondent with the infection rate of examined rodents with tape worm and nematoda, respectively. Distribution of helminth parasites throughout climate zone in Iran and abundance, mean intensity, and range of helminth species found in 253 rodents from different regions of Iran (2014–2015), were shown on Table 5 and 6, respectively.

**Table 2:** Infectivity of captured rodents with endoparasites

	***M. musculus n=76***	***Microtuss.n=70***	***C. elburzensisn=21***	***A. witherbyi n=51***	***M. libycus n=13***	***T. indica n=15***	***A. elater n=6***	***A. amphibius n=1***
No. of infected animal)	38	32	7	24	3	3	1	1
Infection frequency (%)	50	45.7	33.3	47.05	23	20	16.6	100

**Table 3:** Prevalence of cestodes species collected from 253 rodents captured in 11 provinces of Iran

	***M. musculus N (%)***	***M sp. N (%)***	***C. elburzensis N (%)***	***A. witherbyi N (%)***	***M. libycus N (%)***	***T. indica N (%)***	***A. elater N (%)***	***A. amphibius N (%)***	***Total N (%)***
*H. nana*	1(1.3)	-	-	1(1.9)	-	-	-	-	2(0.8)
*H. diminuta*	1(1.3)	-	2(9.5)	4(7.8)	-	1(6.6)	-	-	8(3.1)
*T. taeniaformis* larva	4(5.2)	-	-	1(1.9)	2(15.3)	-	-	-	7(2.7)
*Mesocestoides*	-	-	1(4.7)	-	-	-	-	-	1(0.4)
larva									

**Table 4:** Prevalence of Nematoda species collected from 253 rodents captured in 11 provinces of Iran

	***M. musculus N(%)***	***M. sp N(%)***	***C. elburzensis N(%)***	***A. witherbyi N(%)***	***M. libycus N(%)***	***T. indica N(%)***	***A. elater N(%)***	***A. amphibius N(%)***	***Total rodents N(%)***
*S. obvelata*	20(26.3%)	15(21.4%)	5 (23.8%)	8(15.6%)	-	1(6.6%)	1(16.6%)	1(100%)	51(20.1)
*A. tetraptera*	9 (11.8%)	6(8.5%)	1 (4.7%)	6(11.7%)	-	-	1(16.6%)	-	23(9.09)
*T. muris*	1(1.3%)	-	-	-	-	-		-	1(0.4)
*Capillaria sp*	-	-	-	1(1.9%)	-	-	-	-	1(0.4)
*Physaloptera sp.*	-	-	-	1(1.9%)	-	1(6.6%)	-	-	2(0.7)
*Gongylonema sp.*	1(1.3%)	-	-	-	2(15.3%)	-	-	-	3(1.1)
*N. brasiliensis*	3(3.9%)	4(5.7%)	-	10(19.6%)	-	-	-	-	17(6.7)
*H. polygyrus*	1(1.3%)	8 (11.4%)	-	2(3.9%)	-	-	-	-	11(4.3)

**Table 5:** Distribution of helminth parasites throughout climate zone in Iran

***Species***	***Zone 1***	***Zone2***	***Zone 3***
*S. obvelata*	+	+	+
*A. tetraptera*	+	+	
*T. muris*		+	
*Capillaria* sp.		+	
*Physaloptera* sp.		+	
*Gongylonema* sp.		+	+
*N. brasiliensis*	+	+	
*H.polygyrus*	+	+	
*H. nana*		+	
*H. diminuta*		+	+
*T. taeniaformis* larva		+	
*Mesocestoides larva*			+
*N. neyrai*		+	
*M. moniliformis*		+	

**Table 6:** Abundance, mean intensity, and range of helminth species found in 253 rodents from different regions of Iran (2014–2015)

	***Abundance (%)***	***Mean Intensity***	***Range***
*S. obvelata*	0.4	5.7	2–70
*A. tetraptera*	0.2	10.6	1–70
*T. muris*	0.008	1	1
*Capillaria sp*	0.008	1	1
*Physaloptera sp.*	0.01	1	1
*Gongylonema sp.*	0.02	1	1
*N. brasiliensis*	0.15	3.8	1–18
*H. polygyrus*	0.09	4.3	2–14
*H. nana*	0.01	1	1
*H. diminuta*	0.07	1.1	2–1
*Taenia taenia formis* larva	0.06	1	1
*Mesocestoides* larva	0.008	1	1
*M. moniliformis*	0.008	1	1
*N. neyrai*	0.008	1	1

## Discussion

Our findings gave us an overview on the helminths parasites fauna of rodents in different parts of Iran, with fifteen species of helminths isolated from thirteen rodent species. The diversity in rodent parasites under study points to their adaptability as well as capability of the host to support them.

Our results indicated that examined rodents were more infected with nematodes than cestodes. Nematodes with 8species had the most diversity in our study. We found only one *Moniliformis moniliformis* (Acanthocephala), isolated from *M. libycus*. The least and rare finding was belonged to the *Notocotylus neyrai* (Trematoda), isolated from water vole (*A. amphibius*) for the first time in Iran

### Rodents

Of the thirteen studied genera of rodents, the genus *Mus* was the most prevalent which is concordant to finding (11). The genus *Microtus* and *Apodemus* stayed at the second to third ranks, respectively. Overall 43.08% of the rodents were infected with at least one helminth species. *M musculus* and *A. witherbyi* with the rate of 50% and 47.1% had the maximum parasitic infectivity rate, respectively. Infection with nematodes was higher than cestodes, resembling the result of other reports (11, 17).

The heterogeneous nature of the parasites within the rodents indicates their resilience. It also highlights the capacity of the host to sustain the nutritional, developmental, physiological and behavioral needs of the parasites (18).

### Nematoda Parasites

We found *S. obvelata* as the most prolific and abundant helminth in all studied rodents, particularly in *M. musculus*, but nothing for *M. libycus*. This is in agreement with the previous reports (12, 19) from different parts of Iran. In contrast, our finding is lower than (20) from Mazandaran Province which may be due to a rainy weather and high humidity in this area. *A. tetraptera* was another most frequent species of helminth in *M. musculus* (11.8%) that commonly reported in mouse and wild mouse in Iran and another country (8, 9, 11, 21, 22). In current study, *A. tetraptera* was most abundant in second climate zone but did not find in Khuzestan, this result is consistent with earlier reports (12, 23). The differences in the prevalence of this helminth between second and third climate zone might be associated with climatic conditions especially air temperature, rainfall which strongly affect the distribution of rodent’s species in this area and also directly influence the development and reproduction of parasites carried by them. Seemingly, *A.tetraptera* distribution is in correlation with ecological condition of its hosts, but this should be call for caution owing to insufficient sampling of southern region of Iran.

*Gongylonema* sp. with the prevalence of 15.3% was the most important helminths recovered in the third climate zone. Presence of invertebrates in the environment plays a significant role in maintaining and spreading *Gongylonema* sp. infections in the study area. This is probably due to the severe hot and very dry weather mainly in the summer. *Gongylonema* sp. was found in *M. musculus* and *M. libycus*. Human infections with *Gongylonema* sp. has been reported (24–27) in the world.

*Physaloptera* sp. was observed in *T. indica and A. witherbyi* with the prevalence of 6.6% and 1.9% from Zahedan and Yasuj, respectively. The cockroach can serve as intermediate host of *Physaloptera sp.,* so these rodents might be acquired by feeding on cock roach. Climate changes strongly affect the survival and distribution of the intermediate cockroach host and also directly influence the development and reproduction of parasites carried by them. Although several species of *Physaloptera* have been found in wild rodents around the world (9, 28) but the information about the life cycle and morphological characters of *Physaloptera* was very rare in Iran.

Other recovered nematoda parasite from rodents in this study including *Capillaria* sp., *T. muris*, *N. brasiliensis* and *H. polygyrus. N. brasiliensis* and *H. polygyrus* were commonly reported in different country (5, 29–33) while in Iran only few record were found (9,11,19,20)

In study which performed on wild rodents in Mazandaran*, N. brasiliensis* was found in 2.7% of *M. musculus* and *A. witherbyi* (20). However the rate of infection with this helminth in our study was higher. *H. polygyrus* has been rarely reported in Iran (19).

The most of three species of rodents (*M. musculus*, *A witherbyi* and *Microtus* sp.) infested with these helminths were isolated form first climate zoon with high humidity. Rodents become infected through penetration of L3 stage of *N. brasiliensis* due to sufficient humidity have the main role in contamination.

In this study*,* since only one *Capillaria* (0.4%) was collected form intestine, it was insufficient for the identification of the species.

### Cestodes parasites

*T. taeniaeformis* larva was the most prevalent cestodes species with infection rate of 2.7% in rodents (7 out of 253). Infected species are belonging to the family Muridae, into the two well-known genera from Iranian plateau, *M. musculus* (5.2%) and *A. witherbyi* (1.9%), in captured in second climate zone. Moreover, 15.3% of *M. libycus* that captured in North Khorasan were infected with this cestod.

Prevalence of *T. taeniaeformis* larva compared (11) on *M. musculus* and *R. norvegicus* in second climate zone (Kermanshah) was higher than our finding (4.3%). *C. fasciolari*s were the common helminths in rodent of India and its prevalence was 35.2% (34). *C. fasciolari*s infects cats but utilizes rodents as an intermediate host and the larva develops in or on the liver. Infection in humans is rare and occurs by accidental ingestion of eggs and liver cysts.

In current study, *H. diminuta* and *H. nana* included 3.1% and 0.8% of the collected rodents, respectively. *H. diminuta* were more recovered from *A. witherbyi* who captured from zone 2, while it was not previously reported from *A. witherbyi* in zone1 (20). The rodent tapeworm, *H. diminuta*, is transmitted when an egg is consumed by an arthropod intermediate host such as the cockroach or flour beetle. Once the infected arthropod is eaten by a rodent development into an adult tapeworm occurs within the intestines. Accidental ingestion of infected arthropods by humans results in the development of an adult tapeworm within the intestines and had been distinguished from long time ago in Iran, Italy, Turkey and Spain (33, 35–37). *H. nana* is commonly reported throughout Iran and other countries (38–40). *H. nana* is common helminth parasite that naturally occurs in both man and rodent and plays a significant role in the prevalence of some of the important human parasites (41).

We found only one *C. elburzensis* infested with *Mesocestoides* larva (0.4%) in peritoneum. This larva was reported from *M. persicus* and *M. socialis* (9).

Other recovered parasites in this study were *M. moniliformis* (0.4%), in which belongs to the Phylum Acanthocephala, with zoonotic potential. The rodents’ serves as the definitive host for *M. moniliformis* and humans considered incidental hosts following ingestion of intermediate hosts.

Cases in humans have been reported from Nigeria, Iran and another country (38, 42–47). The results in current study are lower than those (48) who reported 19% from Anuradhapura, Ampara, and Kandy districts, whereas we found it only in one individual of *M. libycus* in Khuzestan with hot and dry climate.

This study records the first data on the *N. neyra*i from the *A. amphibius* from Zanjan province in northwestern Iran. This vole is an aquatic species adopted to the wet habitats on river banks. There is few and rare reports from this trematode of rodents in world (49, 50).

Among 15 species of helminth parasites isolated from captured rodents following species are considered as zoonotic importance; *Gongylonema*, *S. obvelata*, *Capillaria*, *H. diminuta, H. nana*, *Mesocestoides* larva*, T. taeniaformis* larva and *M. moniliformis* in which *S. obvelata* and *H. diminuta* were the most prevalent helminths. In general, 35.5% of all *M. musculus* were infected with zoonotic helminths. Harboring a wide variety of zoonotic parasites by *M. musculus* (51) particularly when the rodent lives nearby the native population residences represents a potential risk to the health of the population.

Control of zoonotic parasites depends on reliable knowledge of their lifecycles, reservoirs, and distribution and transmission patterns in each zoogeographical situation. Parasitic helminths with either free-living stages in their life cycles or indirect transmission (e.g. via vectors) are sensitive to climate changes and other ecological perturbations. Temperature and humidity influence survival of parasite egg, larvae and the distribution and abundance of parasite vectors

## Conclusion

Parasite with direct life cycle was more abundant in first climate zoon while the most recovered parasite in third climate zoon were parasite with indirect life cycle. The majority of rodent’s species and parasite diversity were observed in second climate zoon with cold and high mountainous areas. Further research is needed to determine the role of different helminth species in regulating rodent population dynamics in different climate zone and their effect on the reproduction of different rodent species.
